# Tailoring Intermolecular Interactions Towards High‐Performance Thermoelectric Ionogels at Low Humidity

**DOI:** 10.1002/advs.202201075

**Published:** 2022-04-28

**Authors:** Wei Zhao, Tingting Sun, Yiwei Zheng, Qihao Zhang, Aibin Huang, Lianjun Wang, Wan Jiang

**Affiliations:** ^1^ State Key Laboratory for Modification of Chemical Fibers and Polymer Materials College of Materials Science and Engineering Donghua University Shanghai 201620 China; ^2^ Soochow Institute for Energy and Materials Innovations College of Energy Key Laboratory of Advanced Carbon Materials and Wearable Energy Technologies of Jiangsu Province Soochow University Suzhou 215006 China; ^3^ Institute for Metallic Materials Leibniz Institute for Solid State and Materials Research Dresden (IFW Dresden) Dresden 01069 Germany; ^4^ State Key Laboratory of High Performance Ceramics and Superfine Microstructure Shanghai Institute of Ceramics Chinese Academy of Sciences Shanghai 200050 China; ^5^ Center of Materials Science and Optoelectronics Engineering University of Chinese Academy of Sciences Beijing 100049 China; ^6^ Institute of Functional Materials Donghua University Shanghai 201620 China

**Keywords:** intermolecular interactions, ionic Seebeck coefficient, low humidity, stretchability

## Abstract

Development of ionic thermoelectric (iTE) materials is of immense interest for efficient heat‐to‐electricity conversion due to their giant ionic Seebeck coefficient (*S*_i_), but challenges remain in terms of relatively small *S*_i_ at low humidity, poor stretchability, and ambiguous interaction mechanism in ionogels. Herein, a novel ionogel is reported consisting of polyethylene oxide (PEO), polyethylene oxide‐polypropylene oxide‐polyethylene oxide (P123), and 1‐ethyl‐3‐methylimidazolium acetate (Emim:OAC). By delicately designing the interactions between ions and polymers, the migration of anions is restricted due to their strong binding with the hydroxyl groups of polymers, while the transport of cations is facilitated through segmental motions due to the increased amorphous regions, thereby leading to enlarged diffusion difference between the cations and anions. Moreover, the plasticizing effect of P123 and Emim:OAC can increase the elongation at break. As a consequence, the ionogel exhibits excellent properties including high *S*_i_ (18 mV K^−1^ at relative humidity of 60%), good ionic conductivity (1.1 mS cm^−1^), superior stretchability (787%), and high stability (over 80% retention after 600 h). These findings show a promising strategy to obtain multifunctional iTE materials by engineering the intermolecular interactions and demonstrate the great potential of ionogels for harvesting low‐grade heat in human‐comfortable humidity environments.

## Graphical abstract

By tailoring the component among ionogels, a new paradigm for regulating the intermolecular interaction between ionic liquids and polymer matrix is proposed, which results in high ionic Seebeck coefficient with excellent long‐term stability at low environmental humidity. The dynamic hydrogen bonding interaction enhances thermoelectric properties while endowing the ionogel large elongation at break.

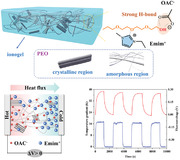

## 1 Introduction

Research into low‐grade heat harvesting and the conversion from environmental or body heat into useful electricity have been stimulated by the energy and environmental crisis as well as the emerging market based on wearable electronics and the Internet of things.^[^ ^1^, ^2^ ^]^ Thermoelectric generation (TEG) based on Seebeck effect shows great potential in directly converting heat into electricity without any moving parts. Recent years have witnessed considerable progress on improving the TE performance for traditional electronic TE materials. However, the Seebeck coefficient of these electronic TE materials (*S*_e_) is usually a few hundred µV K^−1^, leading to very low output voltages.^[^ ^3^ ^]^ Besides, the strong coupling between the Seebeck coefficient, electrical conductivity and thermal conductivity remains a huge obstacle limiting further improvements in TE conversion efficiency.^[^ ^3^ ^]^ In contrast, ionic thermoelectric (iTE) materials, driven by thermal diffusion of ions, have recently received increasing interest owning to their significantly larger ionic Seebeck coefficient (*S*_i_, >1 mV K^−1^).^[^ ^4^, ^5^ ^]^ Different from the electron or hole carriers of electronic TE materials which could be utilized in TE generators from a stable temperature gradient, iTE materials have ionic charge carriers that diffuse under a temperature gradient through the Soret effect. Since the ions cannot pass through the electrodes, the thermodiffused ions can be utilized to charge supercapacitors or batteries directly, providing good opportunities to achieve an “all‐in‐one” device that simultaneously generates and stores electrical energy under intermittent heat sources.^[^ ^6^ ^]^

Existing iTE materials include polyelectroyltes, polyelectrolytes in conducting polymers, ionic liquid (IL)‐based ionogels, and hybrid materials.^[^ ^6^ ^]^ Compared to those iTE materials containing water or organic solvents, IL‐based ionogels are considered superior due to the nonvolatility and high thermal stability of ILs.^[^ ^7^ ^]^ To date, a series of ionogels with high TE properties have been reported, for example, poly(vinylidene fluoride‐cohexafluoropropylene/1‐ethyl‐3‐methylimidazolium dicyanamide (PVDF‐HFP/EMIM:DCA) with *S*_i_ of 26 mV K^−1^,^[^ ^8^ ^]^ SiO_2_/Emim:DCA with *S*_i_ of 14.8 mV K^−1^,^[^ ^7^ ^]^ and waterborne polyurethane/Emim:DCA with *S*_i_ of 34.5 mV K^−1^.^[^ ^9^ ^]^ However, these extremely high *S*_i_ are often obtained at very high relative humidity (RH, e.g., 90%^[^ ^9^ ^]^). Considering that the optimum RH for human health and comfort is 40–60%,^[^ ^10^ ^]^ it is desirable to develop high‐performance iTE materials suitable for low‐RH environments to enable a wider range of applications. Furthermore, for the thermodiffusion based on the Soret effect, it is speculated that the *S*_i_ produced by the difference of anion and cation concentrations on the hot and cold sides is complexly influenced by several parameters such as the mass diffusion coefficient, and the Eastman entropy of transfer related to the interaction between ions and surrounding environment.^[^ ^11^, ^12^, ^13^ ^]^ Nevertheless, a complete understanding of the interactions at the atomic level is lacking.

In addition to humidity, the interaction between polymers and ions in the ionogels also plays a crucial role in the ion transport. Polymers of ethylene oxide have been reported to boost the mobility of cations. For example, in 2003, a Seebeck coefficient of about 2.99 mV K^−1^ has been found in undoped PEO due to mobility of thermally activated positive ions.^[^ ^14^ ^]^ Besides, when polyethylene glycol (PEG, a polymer of ethylene oxide with a molecular mass below 20 000 g mol^−1^) was added into PVDF‐HFP/Emim:TFSI, the *S*_i_ was converted from n type to p type because of the ability of PEG to facilitate the migration of Emim^+^.^[^ ^12^ ^]^ Crispin and co‐workers also reported that polyethylene oxide (PEO, a polymer of ethylene oxide with a molecular mass above 20 000 g mol^−1^) enables the faster transport of Na^+^.^[^ ^15^ ^]^ However, for liquid PEG or PEO, there is a risk of leakage and therefore encapsulation is required. Moreover, the reported ionic conductors based on PEO are nonstretchable, failing to meet the requirements of devices such as wearable electronics.

Given the above issues, it is essential to develop iTE materials that offer a combination of advantages, including quasi‐solid state, non‐volatility, high TE performance properties at ambient humidity, and excellent mechanical properties. Herein, we propose an effective solution that satisfies the above requirements. A novel quasi‐solid iTE material is designed, which consists of PEO with high molecular weight (1 000 000 g mol^−1^), a block copolymer (polyethylene oxide‐polypropylene oxide‐polyethylene oxide, P123), and a IL (1‐ethyl‐3‐methylimidazoliumacetate, Emim:OAC). Different from the traditional concept that utilizes the ion–dipole interactions to increase the ion transport,^[^ ^8^ ^]^ we have effectively tailored the intermolecular interactions in iTE gels. The ether oxygen groups on the PEO chains are utilized to conduct Emim^+^ while the migration of OAC^−^ is inhibited due to their strong binding to the terminal hydroxyl groups provided by PEO and P123, thereby enlarging the diffusion difference between the anions and cations. Moreover, the chain segments are effectively activated in the presence of IL and P123 due to the suppression of crystallization resulting from the plasticizing effect. As a result, this new ternary hybrid exhibits an exceptional *S*_i_ of 18 mV K^−1^ at RH of 60%, which is competitive or even higher than those values for iTE materials at such low humidity. Furthermore, it possesses ultra‐high mechanical stretchability of 780% attributed to the dynamic hydrogen bonding between PEO, P123, and IL. As the thermally diffused ions can directly charge a supercapacitor or a battery, we have fabricated ionic thermoelectric capacitors (iTECs), which demonstrate their competence for energy generation and storage.

## 2 Results and Discussion

### 2.1 Design Principle and TE Properties of PEO‐IL Ionogels

**Figure 1:**
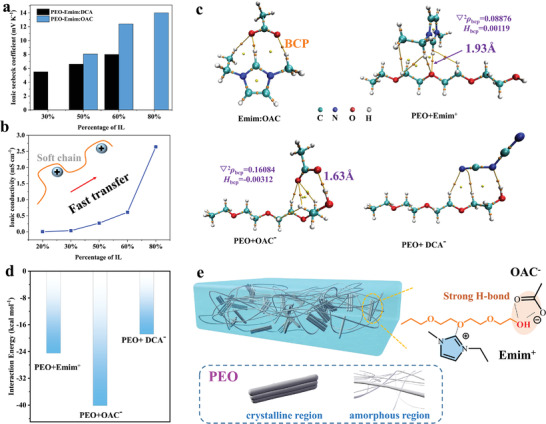
a) Ionic Seebeck coefficient of the PEO‐Emim:OAC and PEO‐Emim:DCA ionogels with varying proportion of IL. b) Ionic conductivity of PEO‐Emim:OAC ionogels with different Emim:OAC contents. c) Configurations of Emim:OAC, PEO+Emim^+^, PEO+OAC^−^, and PEO+DCA^−^ clusters for density functional theory calculations, and d) the corresponding interaction energy between PEO and different ions. e) Schematic illustration and molecular structures of the PEO‐Emim:OAC TE ionogel. The dynamic crosslinking networks are formed through strong hydrogen bonds between OAC^−^ anions and PEO, while weak electrostatic interactions are generated between Emim^+^ cations and PEO.

On one hand, PEO polymers with strong polar networks and large molecular weights have shown excellent absorption of imidazolium‐based ILs due to the formation of hydrogen bonds.^[^ ^16^, ^17^ ^]^ On the other hand, among the imidazolium‐based ILs, Emim:OAC is one of the most commonly used ILs for processing strongly hydrogen bonded materials, such as cellulose,^[^ ^18^, ^19^ ^]^ but few studies have focused on TE applications. Therefore, for the first time, we investigated the TE properties by combining PEO with Emim:OAC, considering that the cations diffusivity of Emim:OAC is higher than that of anions and PEO is able to promote cation transport.^[^ ^20^ ^]^ **Figure** **1**a presents the *S*_i_ of PEO‐Emim:OAC ionogels with different IL content. We can see that the *S*_i_ increases significantly with increasing the weight percentage of Emim:OAC (*W*_IL_/*W*_PEO+IL_), from *S*_i_ = 8 mV K^−1^ for *W*_IL_/*W*_PEO+IL_ = 50% to *S*_i_ = 13.6 mV K^−1^ for *W*_IL_/*W*_PEO+IL_ = 80%, which are higher than that of pristine Emim:OAC (2 mV K^−1^ shown in Figure S1 in the Supporting Information) and PEO (2.99 mV K^−1^ reported^[^ ^14^ ^]^). Another ionic liquid, Emim:DCA, was also used to synthesize the ionogels. In contrast, PEO‐Emim:DCA ionogels show lower *S*_i_ than the samples made with Emim:OAC at the same weight percentage (Figure 1a). In addition, the ionic conductivity (*σ*_i_) of PEO‐Emim:OAC ionogels was measured using impedance spectroscopy (Figure S2, Supporting Information). As shown in Figure 1b, *σ*_i_ is significantly improved from 0.02 to 2.6 mS cm^−1^ when the weight percentage of Emim:OAC increases from 20% to 80%.

In order to elucidate the interaction mechanism between PEO and different ionic liquids, we performed density functional theory (DFT) calculations and adopted the atoms in molecules (AIM) theory. The chemical structures of PEO, Emim:OAC, and Emim:DCA are shown in Figure S3 in the Supporting Information. The interaction configurations between PEO and different ions at different reactive sites were optimized (Figure 1c). The results show that the unsaturated C^2^–H on the imidazole ring acts as a proton donor forming an intermolecular hydrogen bond with the ether oxygen group of PEO, while OAC^−^ as a proton acceptor prefers to bond with the terminal hydroxyl group of PEO. The interaction energy between PEO and OAC^−^ is larger than that between PEO and Emim^+^ as well as PEO and DCA^−^ (Figure 1d), indicating that the OH…O bond is thermodynamically more stable. Besides, the hydrogen bonding length of C^2^–H⋅⋅⋅O is 1.93 Å (Table S1, Supporting Information), larger than that of OH⋅⋅⋅O (1.63 Å), manifesting a weaker interaction of the former. According to the AIM theory, bond critical point (BCP) can prove the existence of the bond, and its topological parameters including the Laplacian of electron density (*∇* ^2^ *ρ*_bcp_) and energy density (*H*_bcp_) can be utilized to analyze the nature of the atomic interaction.^[^ ^21^ ^]^ As a result, both *∇* ^2^ *ρ*_bcp_ and *H*_bcp_ for C^2^–H⋅⋅⋅O are positive values, which further suggests that the weak interaction is attributed to the electrostatic attraction between the ether oxygen groups of PEO and Emim^+^ cations. In contrast, *∇* ^2^ *ρ*_bcp_ > 0 and *H*_bcp_ < 0 for O–H⋅⋅⋅O confirm that the OAC^−^ anions are tightly bound to PEO through the terminal hydroxyl groups.^[^ ^21^, ^22^ ^]^ Based on these results, we can conclude that the increase of *S*_i_ for PEO‐Emim:OAC ionogels is attributed to the enlarged migration difference between Emim^+^ cations and OAC^−^ anions. As illustrated in Figure 1e, the dual effects of ether oxygen group with Emim^+^ and hydroxyl group with OAC^–^ account for the dissociation of IL and restrict the movement of OAC^−^ through stronger binding. Moreover, it has been reported that the transport of cations can be facilitated in the amorphous regions of PEO activated chains through segmental motion.^[^ ^23^ ^]^ The chain segments can be activated effectively in the presence of IL due to the suppressed crystallization (Figure S4, Supporting Information) resulting from plasticizing effect of IL. In principle, increasing the concentration gradient of dissociated ions at the hot‐ and cold‐side electrodes, or enhancing the difference of thermal mobility between anions and cations is favorable to obtain higher *S*_i_. Therefore, experimental and calculated results here provide guidance for further enhancing the *S*_i_ by introducing more terminal hydroxyl groups to trap anions without affecting the free cations and by facilitating cation transport through plasticizing effect.

### 2.2 Further Optimization of TE and Mechanical Properties

Based on the results above, we can deduce that introducing more hydroxyl groups and reducing the crystallinity of PEO are conducive to achieving higher TE properties. Therefore, we subsequently introduced P123 (a symmetric triblock copolymer comprising PEO and poly(propylene oxide)) with low molecular weight of 5800 to further modulate the interaction between the IL and the PEO matrix. It should be noted that, despite the plasticizing effect of IL, excessive addition such as 80 wt% would lead to leakage and the corresponding thermovoltage response time is too long (Figure S5, Supporting Information). Therefore, we fixed the mass fraction of Emim:OAC at 60% and adjusted the ratio of P123 to PEO in follow‐up studies.

**Figure 2:**
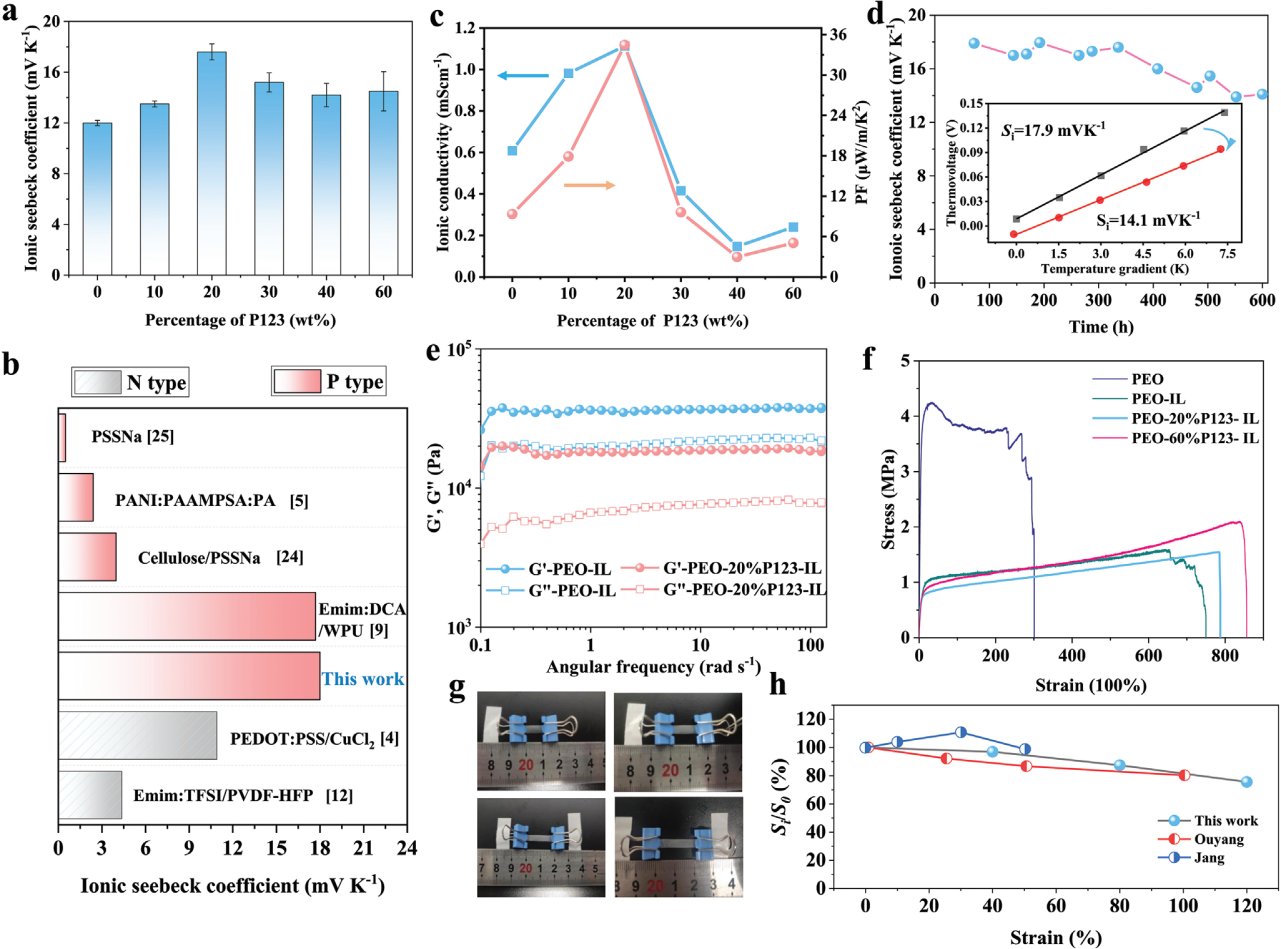
a) Ionic Seebeck coefficient of the PEO‐Emim:OAC ionogels with P123 addition varying from 0% to 60%. b) Absolute ionic Seebeck coefficient of PEO‐20%P123‐IL in this work in comparison with that of reported ionogels measured at relative humidity of 60%.^[^ ^4^, ^5^, ^9^, ^12^, ^24^, ^25^ ^]^ c) Ionic conductivity and power factor of the PEO‐Emim:OAC ionogels with different P123 loading. d) Long‐term performance stability of PEO‐20%P123‐IL ionogel. e) Angular frequency dependencies of the storage modulus (*G*′) and loss modulus (*G*′′). f) Strain–stress curves of pristine PEO and PEO‐based ionogels. g) Photos showing the stretchability of PEO‐20%P123‐IL ionogel in different tensile states and h) the corresponding ionic Seebeck coefficient at different strains. Data from the literature are also included for comparison.^[^ ^5^, ^9^ ^]^

Impressively, with P123 content of 20% the *S*_i_ reaches 18±1 mV K^−1^ (**Figure** **2**a; Figure S6, Supporting Information), a value higher than that of ionogels reported so far at low humidity of 60% (Figure 2b).^[^ ^4^, ^5^, ^9^, ^12^, ^24^, ^25^ ^]^ In addition, we also investigated the effects of humidity and encapsulation on the *S*_i_. As shown in Figure S7 (Supporting Information), the *S*_i_ first increases with the increasing relative humidity, reaching the maximum at relative humidity of 40%, and decreases at higher relative humidity. This is because at lower relative humidity, the absorbed water facilitates the dissociation and diffusion of cations, contributing to higher S_i_. Nevertheless, higher relative humidity (e.g., 80%) leads to sticky surface and even disintegration of the sample, which deteriorates the properties. Hence, the ionogels in this work are suitable for use in low humidity environment. In addition, we found that the encapsulation has little effect on the magnitude of *S*_i_ in our materials at low humidity. The thermovoltages generated under temperature gradient are almost the same in the case of encapsulation and unencapsulation when the relative humidity is not higher than 60%. These results exclude the previously reported influence of hydrovoltaic voltage on the ionic Seebeck coefficients.^[^ ^26^ ^]^

Further increase of P123 content results in lower *S*_i_, but it is still higher than that in the absence of P123. At the same time, *σ*_i_ increases dramatically, from 0.6 to 1.1 mS cm^−1^ when P123 increases from 0 to 20 wt% (Figure 2c; Figure S8, Supporting Information). Consequently, the maximum power factor (PF) of ≈33 µW m^−1^ K^−2^ is achieved using 20 wt% P123 (referred to as PEO‐20%P123‐IL). Moreover, we investigated the thermal voltage reponse of PEO‐20%P123‐IL subjected to different temperatures gradients (Δ*T*). As a result, the thermal voltage displays the linear dependence on the given temperature gradient with a fixed ratio (Figure S9, Supporting Information). The thermal voltage still maintains a excellent linear connection when Δ*T* changes from negative to positive and the open‐circuit voltage is zero when the Δ*T* is zero, indicating that there are no other effects that kicks in, such as interfacial effects. When the Δ*T* increases from 0.7 to 16 K, the corresponding ratio remains between 17 to 18 (Figure S9c–f, Supporting Information), which is consistent with the *S*_i_ and also indicates an excellent temperature sensitivity of the ionogel. The thermovoltages of ionogels were also measured during the repeated heating and cooling cycling tests when exposed to a small Δ*T* of 0.7 K and a large Δ*T* of 16 K (Figure S10, Supporting Information), which show good reproducibility. In addition, to assess the stability of our ionogels, we stored the samples in a laboratory with 60% relative humidity and measured the *S*_i_ of the samples after different storage time. It can be observed that almost 80% of the original *S*_i_ has been retained after over 600 h (Figure 2d), demonstrating the excellent durability and long‐term stability of the ionogel. This is attributed to the nonvolatility and high stability of the ILs, avoiding the degradation of TE properties due to solvent evaporation.

In addition to regulating the thermoelectric properties, we found that the addition of P123 also affects the mechanical properties of the ionogels. The storage modulus (*G*′) and loss modulus (*G*′′) of PEO‐60wt%Emim:OAC (referred to as PEO‐IL) and PEO‐20%P123‐IL were characterized by dynamic mechanical measurements. The *G*′ is higher than *G*′′ (Figure 2e), indicating the quasi‐solid gel behavior. Moreover, both *G*′ and *G*′′ of PEO‐20%P123‐IL are lower than those of PEO‐IL. This means that the PEO‐20%P123‐IL ionogel is softer and P123 can act as a plasticizer. Tensile measurements were also conducted. It can be seen from Figure 2f that pure PEO film without IL possess high Young's modulus of 76.8 MPa and an elongation at break of 300%, which is due to the heavy entanglements of polymer chains and abundant crystalline region.^[^ ^27^ ^]^ After the addition of IL and P123, the tensile strength decreases while the stretchability significantly increases. Notably, the ionogel composed of PEO with 20 wt% P123 and 60 wt% Emim:OAC exhibits superior stretchability with an elongation at break of 787%. Furthermore, we investigated the change of *S*_i_ under different stretched state. As a result, the *S*_i_ maintains well when the ionogel works at strains of up to 120% (Figure 2g,h). Compared to previous reports,^[^ ^5^, ^9^ ^]^ our ionogel shows a more satisfactory retention of the TE properties under stretching (Figure 2h).

### 2.3 Interaction Mechanisms Among Emim:OAC, PEO, and P123

**Figure 3:**
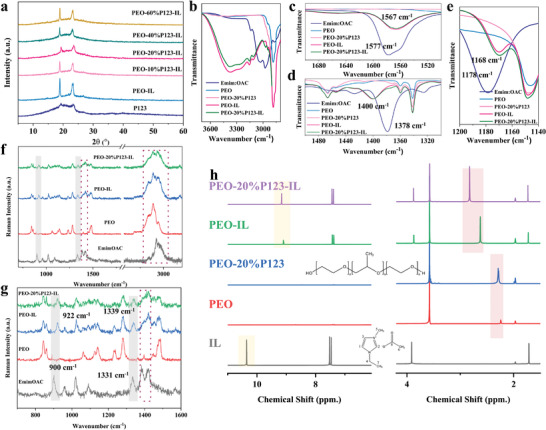
a) XRD patterns of P123, PEO‐Emim:OAC and PEO‐Emim:OAC with different content of P123. ATR‐FTIR spectra of different samples in the range of b) 3700 and 2800 cm^−1^, c) 1700 and 1500 cm^−1^, d) 1500 and 1300 cm^−1^, and e) 1200 and 1140 cm^−1^. f,g) Raman spectra and h) ^1^H NMR spectra.

Both TE and mechanical properties of the PEO‐IL ionogels have been significantly improved due to the addition of P123. In order to investigate the interaction among IL, PEO, and P123, we conducted detailed structural characterization. XRD patterns in **Figure** **3**a show that there are two strong diffraction peaks located at 19.18° and 23.36°, indicating the semicrystalline nature of PEO. Their intensity decreases with the addition of P123 into PEO‐IL, suggesting the increase of the amorphous region. As a result, chain segmental motion can be activated due to the reduction in the energy barrier.^[^ ^28^ ^]^ DSC was utilized to investigate the compatibility and plasticizing effect of P123 on the PEO‐IL ionogels. Figure S11a in the Supporting Information displays a general decrease in melting point with increasing P123 content. However, a new endothermic peak appears around 30 °C when the mass ratio of P123 exceeds 20% (Figure S11b, Supporting Information), suggesting the poor compatibility of P123 with the ionogels at high loading. To sum up, the introduction of P123 with low content can effectively decrease the crystallization of PEO, contributing to the enhancement of *S*_i_ and *σ*_i_.

Figure 3b and Figure S12 (Supporting Information) show the ATR‐FTIR spectra of different samples. The stretching vibration of C^2^–H of Emim^+^ in pure IL is observed at around 3010 cm^−1^. But it disappears after mixing with PEO and P123, and there appears a new broad peak at 3400 cm^−1^. This indicates the existence of intermolecular hydrogen bonds and the dissociation of the originally complexed cations and anions, which enable more Emim^+^ to be transported as the main charge carriers driven by the temperature gradient.^[^ ^29^, ^30^ ^]^ The bands of Emim:OAC at 1577 and 1378 cm^−1^ (Figure 3c,d, Supporting Information) correspond to the symmetric and antisymmetric stretching vibrations of OAC^−^. The difference between the two bands decreases from 199 to 164 cm^−1^, which can be explained by the coordination change of the anions.^[^ ^18^ ^]^ In addition, the C–O stretching band of OAC^−^ at 1178 cm^−1^ in pure IL is shifted to 1168 cm^−1^ in the PEO‐based ionogels (Figure 3e), confirming the formation of COO⋅⋅⋅PEO(OH) hydrogen bonds.^[^ ^31^ ^]^ In the Raman spectra (Figure 3f), the intensity of the peaks at 843 and 861 cm^−1^ corresponding to the C–O stretching and C–H rocking vibration of the representative crystalline PEO is reduced due to the addition of IL and P123. This demonstrates a reduction in the crystallinity of PEO,^[^ ^32^ ^]^ which is consistent with the XRD results discussed above. Passerini et al. pointed out that this phenomenon results from the weakening of the C–O bonding due to the coordination of cations with PEO.^[^ ^33^ ^]^ Accordingly, the peak at 3180 cm^−1^ corresponding to the C–H stretching mode of cations shows slight a shift to 3170 cm^−1^.^[^ ^34^ ^]^ Furthermore, typical peaks for the acetate anions are recorded at 900 and 1331 cm^−1^ (Figure 3g), which is associated with the mixed character of O–C–O bending with C–C stretching vibrations and symmetric C–O stretching vibrations.^[^ ^35^ ^]^ For the ionogels, these two peaks are red‐shifted to 922 and 1337 cm^−1^, respectively, implying the formation of strong hydrogen bonds by the anions. Therefore, the interaction between the cations and anions has been disrupted.

In order to directly prove the role of hydroxyl groups in the ionogels, we then performed the ^1^H NMR measurements (Figure 3h). The positions of H on Emim:OAC and PEO are marked according to the published reports.^[^ ^20^, ^36^ ^]^ There are no additional resonance lines for the ionogels, which implies that the cations and anions are stable and no new species are formed. However, the ^1^H resonance peak of C^2^–H in the PEO‐IL ionogels and PEO‐20%P123‐IL ionogels shifts from 10.3 to 9.2 ppm due to hydrogen bonding effects, while the peaks attributed to other H of Emim^+^ show a slight shift. This indicates that the main action site is on C^2^–H. Additionally, a small resonance peak of the terminal hydroxyl group in PEO is detected at 2.2 ppm. It becomes stronger due to the addition of P123 which has more hydroxyl groups. When we used D_2_O as the solvent, the peak disappears from the spectrum (Figure S13, Supporting Information), confirming that the peak is attributed to the hydroxyl group. Consequently, it moves towards lower fields in the ionogels. This stems from the fact that as the extranuclear electron cloud density of the hydrogen atom forming the hydrogen bond decreases, the shielding effect on the external magnetic field is weakened, resulting in a higher chemical shift of the hydrogen atom.^[^ ^37^ ^]^ The highest chemical shift of OH can be observed in the PEO‐20%P123‐IL ionogel, suggesting the stronger coordination of OH with anions, which is consistent with the theoretical calculations above.

Taken together, the coordination effect of PEO with anions and cations disrupts the interaction between the anions and cations and facilitates the independent migration of the ions. The more terminal hydroxyl groups provided by P123 bind tightly to the anions, restricting the migration of anions. The enlarged diffusion difference between ions thereby effectively increases the ionic Seebeck coefficient. In addition, the P123 also acts as a plasticizer to make the polymer chain motion easier, which boosts the cation mobility and thus leads to satisfactory *S*_i_ at low RH. Meanwhile, due to the plasticizing effect, the ionic conductivity and the stretchability are both significantly improved.

### 2.4 Demonstration of Ionic Thermoelectric Capacitors

**Figure 4:**
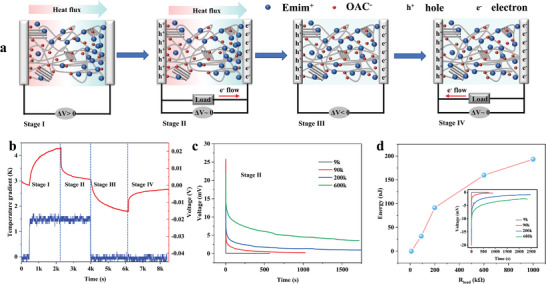
a) Schematic illustration of the energy conversion and storage principles of ionic thermoelectric capacitors. b) Thermovoltage profiles with an external load connected or disconnected under given temperature gradients. c) Thermovoltage curves under different external loads at stage II. d) Generated energy at different external loads. The inset shows thermovoltage curves under different external loads at stage IV.

Considering the thermocapacitive behavior of ionogels, where the thermovoltage between the cold and hot sides is generated by ion diffusion based on the Soret effect, ionic thermoelectric capacitors (iTECs) were assembled with PEO‐20%P123‐IL and Ag electrodes. The specific capacitance of the iTEC is 52.7 µF cm^−2^ according to the cyclic voltammogram (CV) measured at a scan rate of 200 mV s^−1^ (Figure S14a, Supporting Information). It shows a near rectangular shape, revealing a typical capacitive behavior.^[^ ^38^ ^]^ **Figure** **4**a illustrates the working mechanism of the iTECs. The corresponding thermovoltage dependence on given temperature gradient and external loading is shown in Figure 4b. There are four stages in one thermal cycle. At stage I, when a temperature gradient of 1.4 K is applied to the ionogel, an open‐circuit voltage of ≈23 mV is generated resulting from the diffusion and accumulation of ions on the hot‐ and cold‐side electrodes. An external load is subsequently connected at stage II, which results in the decay of thermovoltage approaching to zero. This is because electrons flow through the external circuit and neutralize the charge imbalance on the electrodes. When the temperature gradient is removed and the external load is disconnected at stage III, the ions accumulated on the electrodes gradually return to their original random state while the electrons left on the cold side generate a reverse voltage, which confirms that electrons and holes have been transferred to the electrodes. Finally, the external load is reconnected, leading to the decrease approaching zero in reverse voltage charged at stage II. Using Ag wires as electrodes, the charging and discharging time at stages II and IV display great dependence on the external load (Figure 4c,d), where the decay time is proportional to the product of electrode capacitance and the load resistance. During these processes, there is a self‐discharge behavior, which is reflected by the decay of open voltage after electrical charging (Figure S14b, Supporting Information). Similar phenomena were also reported for iTECs using PEO‐NaOH electrolyte and SiO_2_‐IL ionogel.^[^ ^15^, ^39^ ^]^ To address this issue, more efforts need to be devoted to capacitor research, such as optimizing the device configuration and developing electrodes with higher specific surface area.

Herein, the energy harvested from both stage II and IV can be calculated as follows^[^ ^9^ ^]^

(1) $$\mathrm{Energy}=\int U^{2}/Rdt$$

where *U* and *R* are the output voltage and external load resistance, respectively. Figure 4d and Figure S15 (Supporting Information) show that the harvested energy increases with enlarging external resistance over the measurement range of 9 kΩ to 1 MΩ. As a result, a total energy of 193.2 nJ can be achieved when the ionogel is connected with a load of 1 MΩ at a temperature gradient of 1.4 K. We also investigate the voltage profile on an external load of 200 kΩ connected to the iTEC under different temperature gradients and measured the energy harvested from the electrical charging stage II. The harvested energy increases quadratically with temperature gradient (Figure S16, Supporting Information), confirming the thermally induced charging behavior.

## 3 Conclusion

Quasi‐solid state ionogels consisting of PEO, Emim:OAC and P123 have been developed in this work, which exhibit superior ionic Seebeck coefficient of 18 mV K^−1^ at 60% RH and possess excellent stretchability and stability. Density functional theory calculations and detailed structural characterization were carried out and the interaction mechanism between different components have been fully elucidated. Ionic thermoelectric capacitors have been assembled, which has demonstrated the benefits of simultaneous thermoelectric conversion and energy storage. Our results provide a new paradigm for regulating the intermolecular interaction between ionic liquids and polymer matrix, and broaden the research vision for developing high‐performance iTE materials, which will accelerate the implementation of iTE technology to alleviate the energy and environmental crisis.

## 4 Experimental Section

### Materials and Chemicals

PEO with an average weight molecular of 1 000 000 g mol^−1^ was bought from Alfa, and P123 with an average weight molecular of 5800 g mol^−1^ was bought from Sigma‐Aldrich. Emim:OAC and Emim:DCA were provided by Lanzhou Greenchem ILS, LIPC, CAS (Lanzhou, China). Acetonitrile (A.R.) was supplied by Sinopharm Chemical Reagent Co., Ltd. All of the reagents were used as received without any further purification.

### Sample Synthesis

The ionogels were synthesized by mixing Emim:OAC or Emim:DCA with acetonitrile solution of PEO or the mixture of PEO and P123 in different weight ratios. For the mixture of IL and PEO, the weight ratio of IL varied from 20% to 80%. In the ternary composition, the content of P123 among the total weight of polymers has been regulated in the range of 10% to 60% while keeping the *w*_IL_:*w*_polymer_ = 3:2. The mixture was magnetically stirred for 12 h until being homogeneous and then casted into a glass mold. Finally, the freestanding and flexible ionogels were obtained by drying in a fume hood overnight followed by in a vacuum oven at 60 °C for 12 h.

### Characterization

*S*_i_ measurements were conducted by a homemade equipment based on the definition as $S_{i}=-\frac{\Delta V}{\Delta T}$. Ag wire (0.25 mm, 99.9985%) was used as the electrode. The open circuit voltage change Δ*V* was collected by a nanovoltmeter (Keithley 2182A) and temperature change data Δ*T* was monitored via an infrared camera (FOTRIC 226). To ensure the accuracy of the measurement, the *S*_i_ of Emim:OAC was measured using the same method via this equipment. The result was 2.2 mV K^−1^, which is consistent with the reported value.^[^ ^40^ ^]^ The ionic conductivity *σ*_i_ was calculated as follows: $\sigma_{i}=\frac{d}{A}\;\frac{1}{R}$, where *d*, *A*, and *R* represent the thickness, area, and ionic resistance, respectively. *R* can be measured using electrochemical impedance spectroscopy (EIS) on electrochemical workstation (DH7006) with the frequency ranging from 0.1 to 10^5^ Hz. The ionogel film was sandwiched between two stainless steel sheets during the measurement. The thickness of the film was about 200 µm as measured by SEM and micrometer. Measurements were performed at least five times of five samples to obtain an average value.

The stress–strain tests were conducted using EUT4103 testing instrument with a speed of 3 mm min^−1^ at 25 °C. The rheological properties of ionogels were evaluated using an AR‐G2 rheometer (TA Instrument) with the plate‐to‐plate configuration. Surface morphology and the cross section were characterized via field‐454 emission scanning electron microscopy FE‐SEM (su8000, Hitachi). X‐ray diffraction (XRD, Bruker D8 Discovery A25) was utilized to evaluate the crystallinity of PEO based ionogels in the 2*θ* range from 5° to 60° with a step size of 0.03°. DSC experiments were done by using a Netzsch Phoenix DSC 214. The Fourier transform infrared spectroscopy (FTIR) was performed with Nicolet6700 with the ATR (Attenuated Total Reflection) accessory and Raman spectra were recorded on an InVia Reflex Renishaw spectrometer using the 785 nm laser wavelength.

### Computational Details

The structures of ionic liquid including Emim:OAC and Emim:DCA and their interaction configurations (PEO with Emim:OAC and Emim:DCA) at different reactive site were optimized by using the density functional theory (DFT) at the *ω*B97XD/def2‐SVP level in vacuum by using the Gaussian 16 package, revision A. 01. The harmonic frequency calculations were carried out at the same level of theory to obtain enthalpy, Gibbs free energy, and help verify that all structures have no imaginary frequency.

## Conflict of Interest

The authors declare no conflict of interest.

## Supporting information

Supporting Information

## Data Availability

The data that support the findings of this study are available from the corresponding author upon reasonable request.
