# Supplementary material for: Tailoring Intermolecular Interactions Towards High‐Performance Thermoelectric Ionogels at Low Humidity
Source: Adv Sci (Weinh). 2022 Apr 28;9(20):2201075. doi: 10.1002/advs.202201075 (PMC9284173; doi:10.1002/advs.202201075)
Supplement: Supplementary file 1 — Supporting Information [file ADVS-9-2201075-s001.pdf]

ADVANCED SCIENCE
Open Access

Supporting Information

for *Adv. Sci.*, DOI 10.1002/advs.202201075

Tailoring Intermolecular Interactions Towards High-Performance Thermoelectric Ionogels at Low Humidity

Wei Zhao, Tingting Sun, Yiwei Zheng, Qihao Zhang, Aibin Huang, Lianjun Wang* and Wan Jiang**

Supporting Information

Tailoring intermolecular interactions towards high performance thermoelectric ionogels at low humidity

Wei Zhao[1,#], Tingting Sun[1,#], Yiwei Zheng[2], Qihao Zhang[3], Aibin Huang[4,5], Lianjun Wang[1*], Wan Jiang[1,6*]*

[1] State Key Laboratory for Modification of Chemical Fibers and Polymer Materials, College of Materials Science and Engineering, Donghua University, Shanghai 201620, China

[2] Soochow Institute for Energy and Materials Innovations, College of Energy, Key Laboratory of Advanced Carbon Materials and Wearable Energy Technologies of Jiangsu Province, Soochow University, Suzhou 215006, China.

[3] Institute for Metallic Materials, Leibniz Institute for Solid State and Materials Research Dresden (IFW Dresden), Dresden 01069, Germany

[4] State Key Laboratory of High Performance Ceramics and Superfine Microstructure, Shanghai Institute of Ceramics, Chinese Academy of Sciences, Shanghai 200050, China

[5] Center of Materials Science and Optoelectronics Engineering, University of Chinese Academy of Sciences, Beijing 100049, China

[6] Institute of Functional Materials, Donghua University, Shanghai 201620, China

* To whom correspondence should be addressed:

wanjiang@dhu.edu.cn; wanglj@dhu.edu.cn; q.zhang@ifw-dresden.de

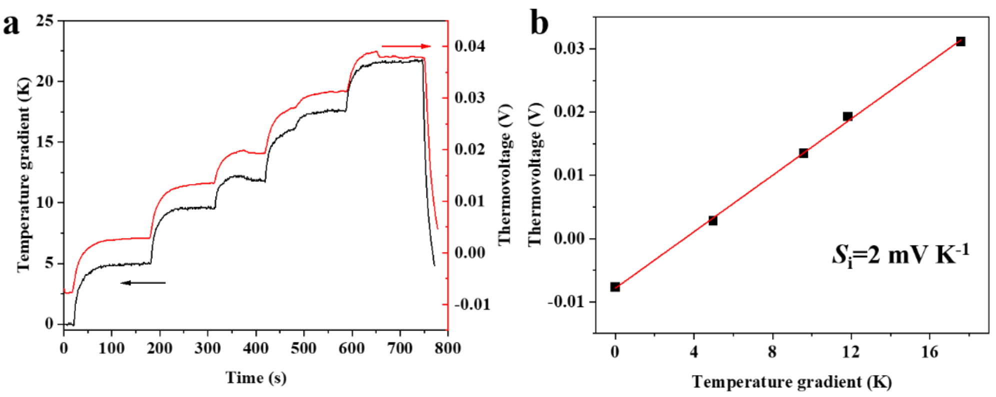

Figure S1. Ionic seebeck coefficient of pure Emim:OAC. a. Measured temperature gradient and corresponding thermovoltage as function of time. b. Linear fitting of thermovoltage with temperature gradient.

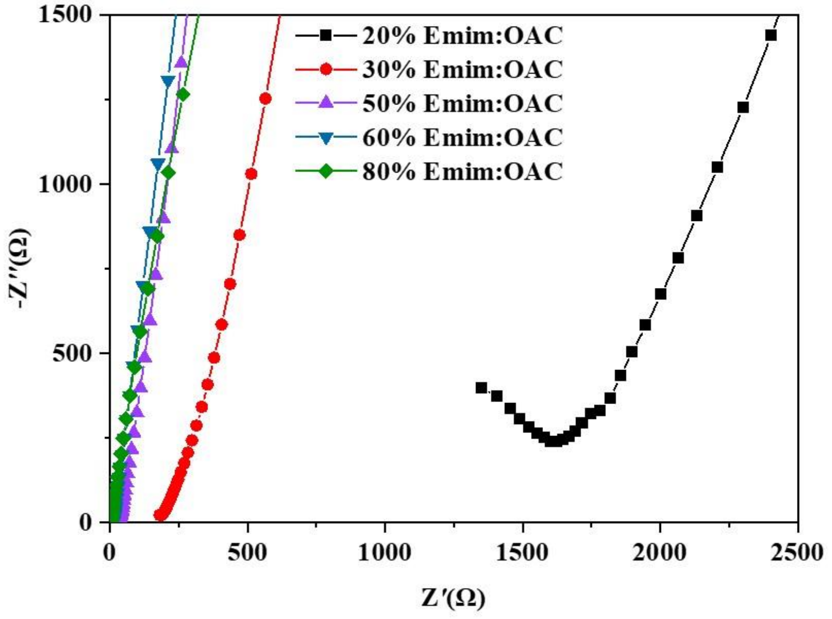

Figure S2. Electrochemical impedance spectroscopy characterization of the PEO-based ionogels with varying IL mass ratio, where the Nyquist plot was recorded.

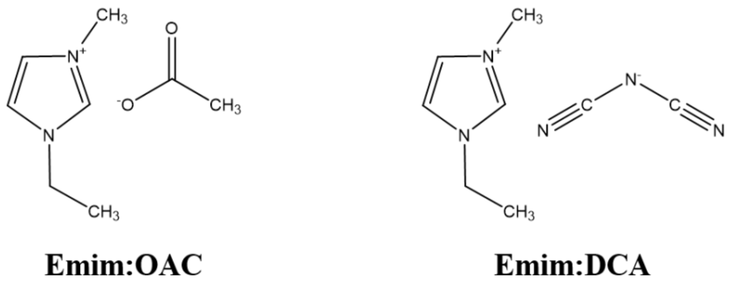

Figure S3. Chemical structure of Emim:OAC, Emim:DCA and PEO.

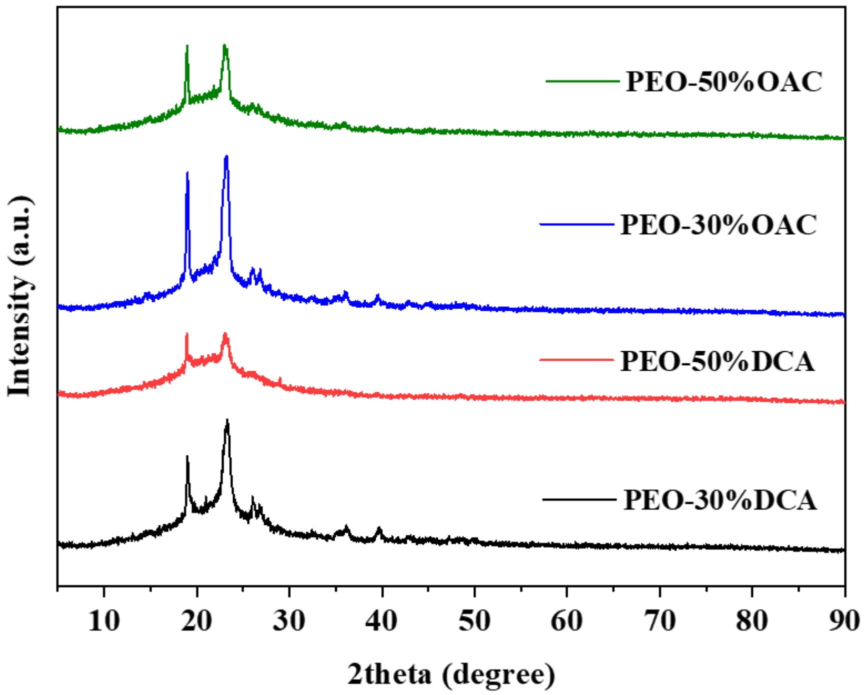

Figure S4. XRD results of PEO-Emim:OAC and PEO-Emim:DCA ionogels with varying IL mass ratio to PEO, indicating that the crystallization of PEO decreases with increasing ionic liquids.

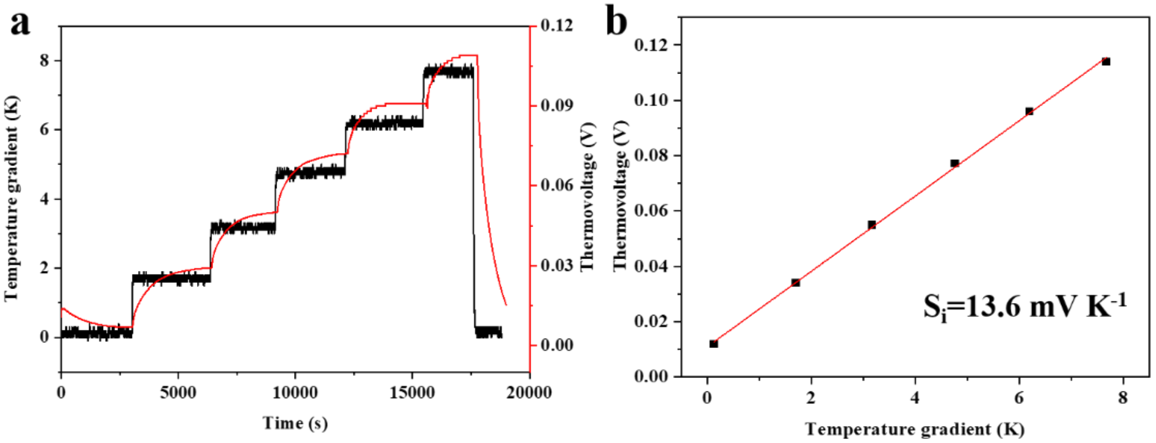

Figure S5. Ionic seebeck coefficient of PEO-80% Emim:OAC. a. Measured temperature gradient and corresponding thermalvoltage as function of the time. b. Linear fitting of thermovoltage with temperature gradient.

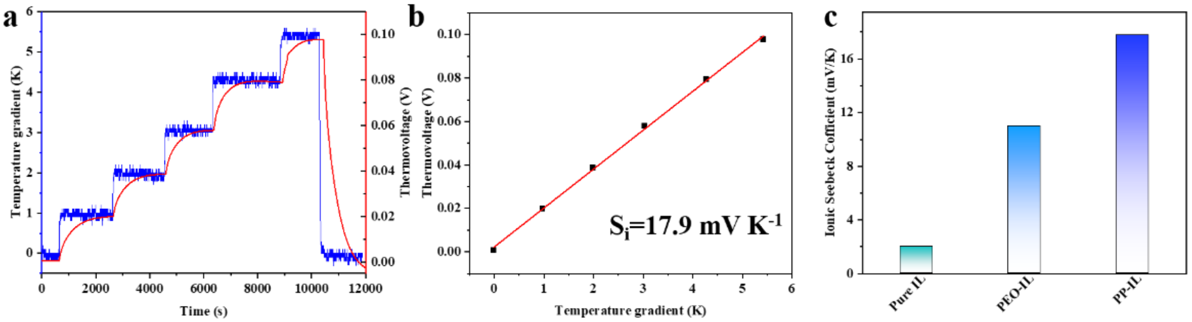

Figure S6. a. Thermovoltage changing with temperature gradient. b. Linear fitting of thermovoltage with temperature gradient of PEO-20wt%P123-60wt%Emim:OAC (referred to as PEO-20%P123-IL). c. Ionic seebeck coefficients for pure Emim:OAC (pure IL), PEO-60wt%Emim:OAC (PEO-IL) and PEO-20%P123-IL.

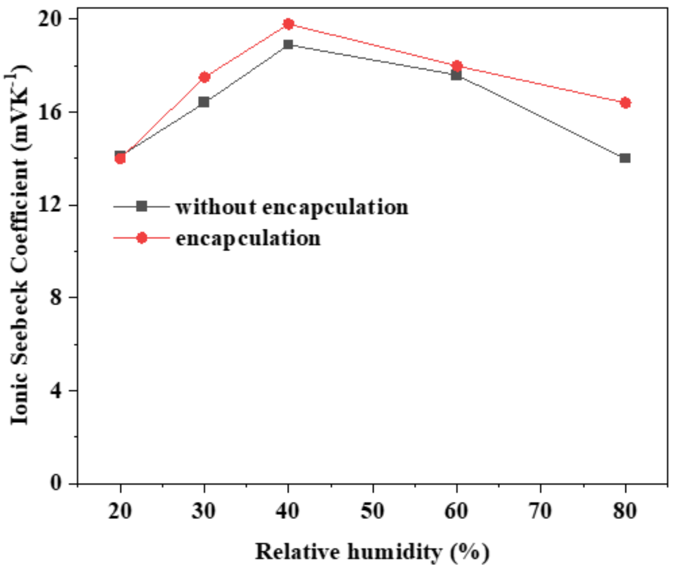

Figure S7. Ionic Seebeck coefficient of the ionogels at different humidity levels. During the experiments, the samples were firstly exposed to different relative humidity, and then encapsulated by polyimide tapes before measurements. For comparison, the measurements were also performed on the samples without encapsulation.

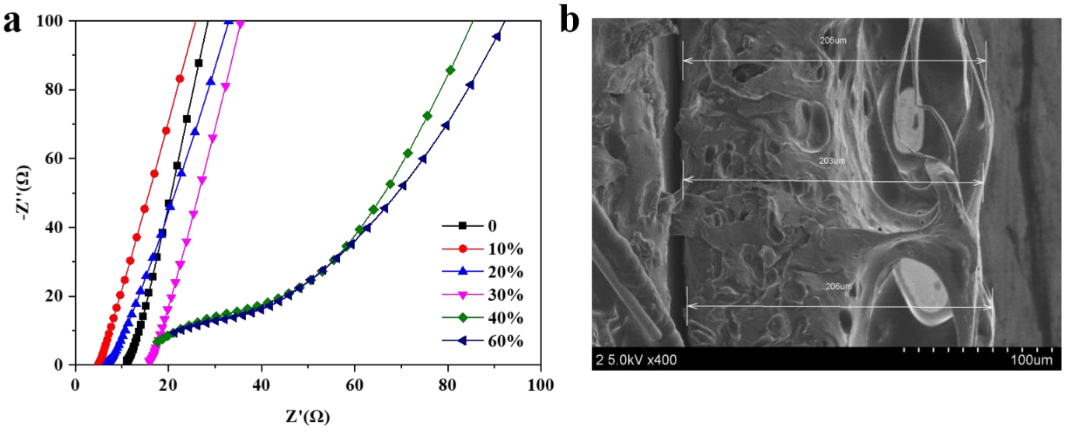

Figure S8. a. Nyquist plot of the PEO-based ionogels with varying P123 mass ratio to PEO. b. Cross-sectional SEM image of PEO-20%P123-IL ionogel film.

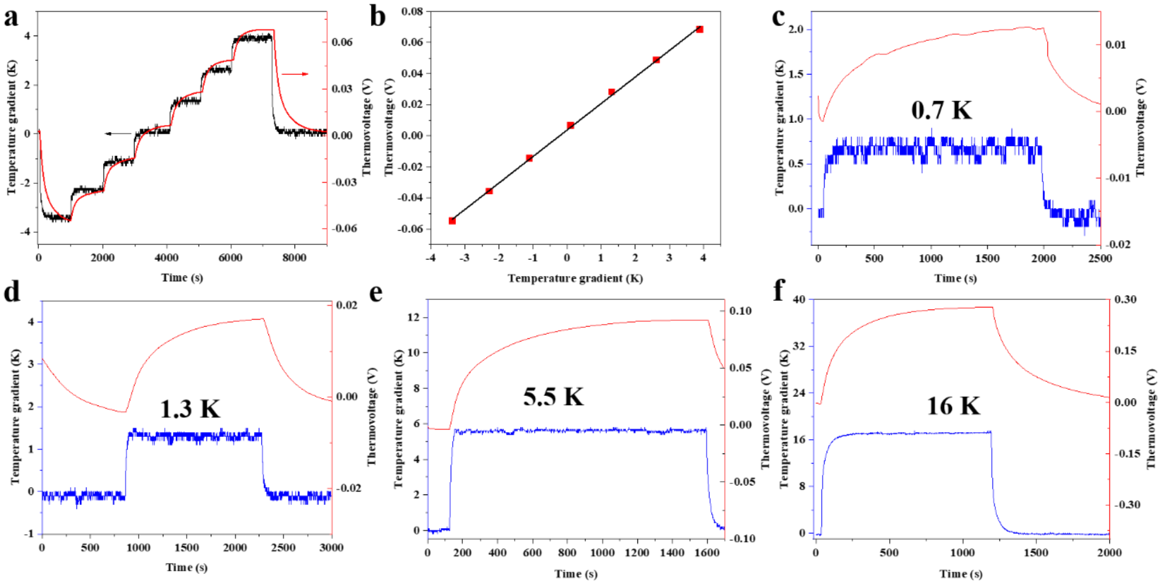

Figure S9. Thermovoltage response curves under different temperature gradients of PEO-20%P123-IL ionogel film.

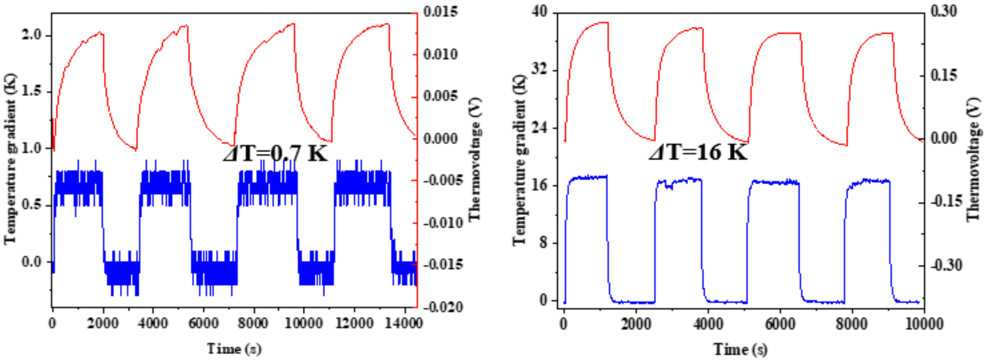

Figure S10. Repeated thermoelectric performance of the PEO-20%P123-IL ionogel against on-off cycles of temperature gradient fixed at 0.7 K and 16 K.

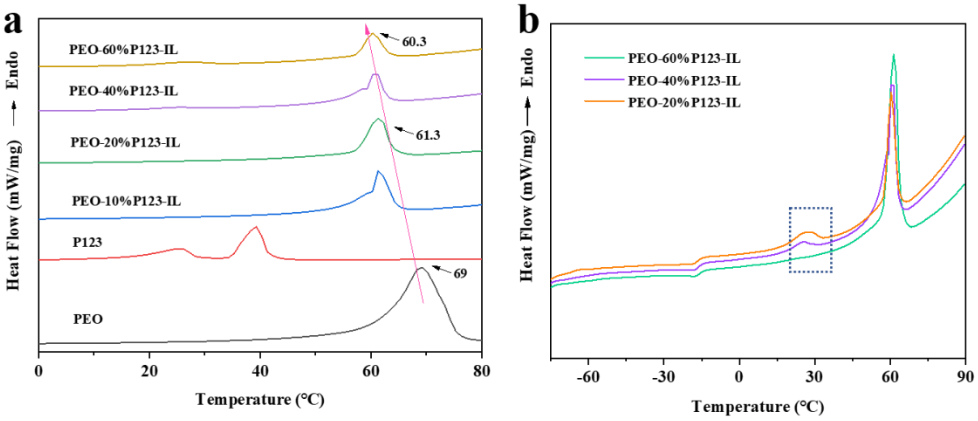

Figure S11. DSC curves of PEO-P123-IL ionogels with increasing weight ratio P123 to PEO.

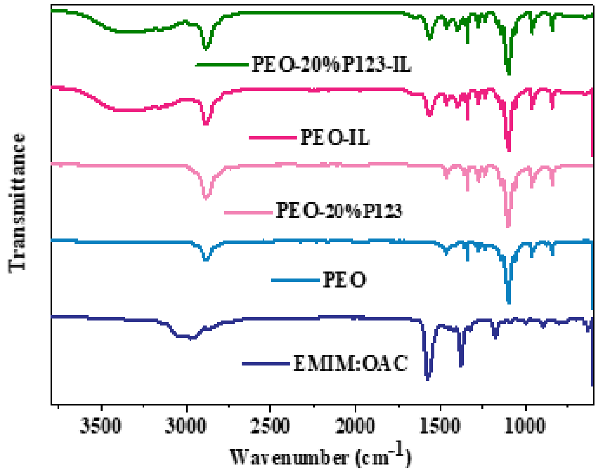

Figure S12. ATR-FTIR spectra of Emim:OAC (IL), PEO, PEO-P123 and the ionogels between 3800 and 600 cm^{-1}.

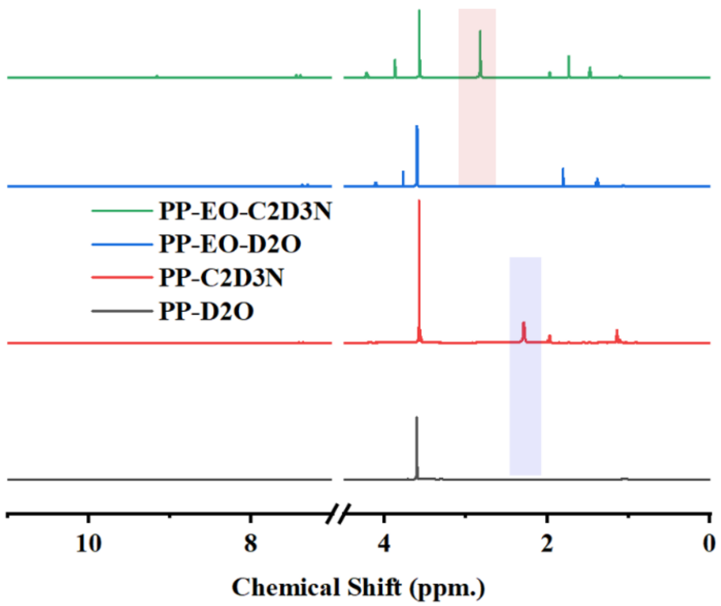

Figure S13. ^{1}H NMR spectra of PEO-20%P123 and PEO-20%P123-IL with C_2D_3N and D_2O as the solvent, respectively.

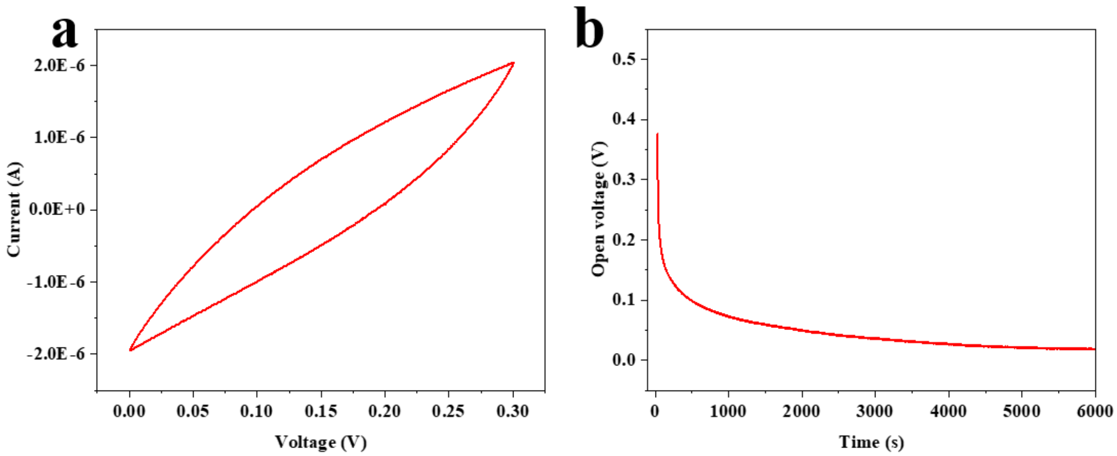

Figure S14. a) Cyclic voltammograms of an ionic thermoelectric capacitor with Ag wire electrodes. The scan rate was 200 mVs^{-1}. b) Decay of open voltage after electrical charging at 0.4 V for capacitor based on Ag electrodes.

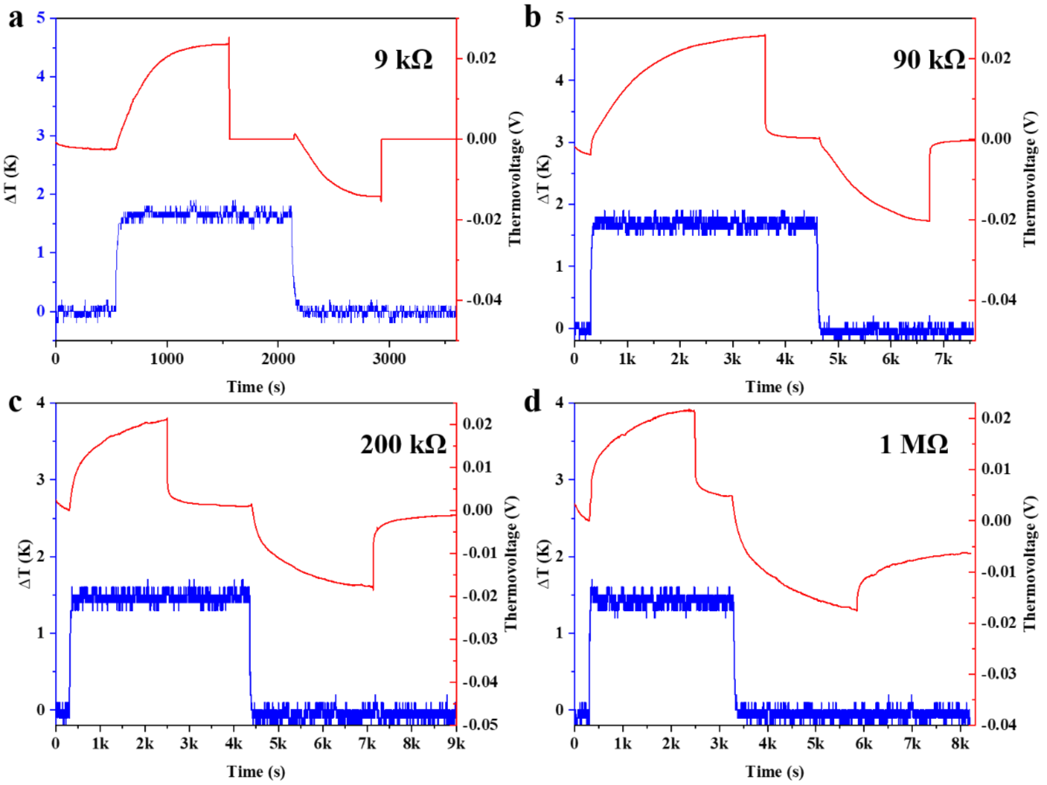

Figure S15. Thermovoltage and temperature gradient curves when connecting or disconnecting an external resistance load varying from 9 kΩ to 1 MΩ.

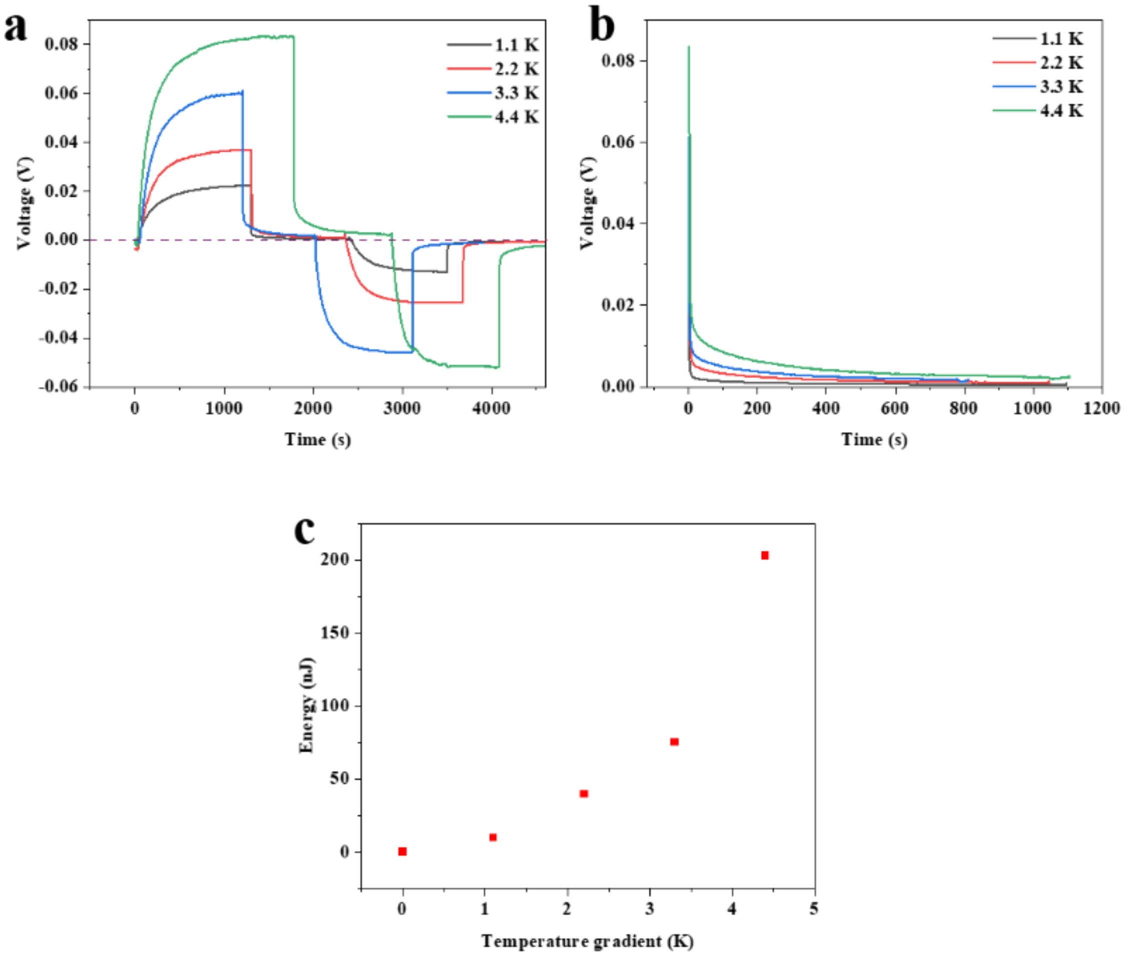

Figure S16. a) Voltage profile on an external load of 200kΩ connected to the iTEC under different temperature gradients. b) Corresponding voltage decay at stage Ⅱ. c) Energy harvested from the electrical charging stage Ⅱ as function of temperature gradient.

Table S1. The calculated hydrogen bond length (*L*), electron density (ρ_{bcp}), Laplacian of electron density ($\nabla^2\rho_{bcp}$), and total energy density (H_{bcp}) at the BCP for the specific intermolecular hydrogen bonds in the PEO-Emim:OAC ionogel.

H-bonds	L	ρ_{bcp}	$\nabla^2\rho_{bcp}$	H_{bcp}
C-H···O	1.93	0.02802	0.08876	0.00119
O-H···O	1.63	0.05303	0.16084	-0.00312
